# Src, PKCα, and PKCδ are required for αvβ3 integrin-mediated metastatic melanoma invasion

**DOI:** 10.1186/1478-811X-7-10

**Published:** 2009-04-28

**Authors:** Andrew J Putnam, Veronique V Schulz, Eric M Freiter, Heather M Bill, Cindy K Miranti

**Affiliations:** 1 Laboratory of Integrin Signaling and Tumorigenesis, Van Andel Research Institute, Grand Rapids, Michigan 49503, USA; 2 Department of Chemical Engineering, University of California, Irvine Irvine, California 92697, USA

## Abstract

**Background:**

Integrins, cell-surface receptors that mediate adhesive interactions between cells and the extracellular matrix (ECM), play an important role in cancer progression. Expression of the vitronectin receptor αvβ3 integrin correlates with increased invasive and metastatic capacity of malignant melanomas, yet it remains unclear how expression of this integrin triggers melanoma invasion and metastasis.

**Results:**

Two melanoma cell lines C8161.9 and M14 both express high levels of αvβ3 integrin and adhere to vitronectin. However, only the highly metastatic C8161.9 cells are capable of invading vitronectin-enriched Matrigel in an αvβ3-depenent manner. Elevated levels of PKCα and PKCδ, and activated Src were detected specifically in the highly metastatic melanoma cells, but not in the low metastatic M14 cells. Inhibition of Src or PKC activity suppressed αvβ3-dependent invasion. Furthermore, over expression of Src or PKCα and PKCδ was sufficient to confer αvβ3-dependent invasiveness to M14 cells. Stress fiber formation and focal adhesion formation were almost completely absent in C8161.9 cells compared to M14 cells. Inhibition of Src signaling was sufficient to restore normal actin architecture, and resulted in decreased p190RhoGAP phosphorylation and enhanced RhoA activity. Src had no effect on Rac activity. Loss of PKCα expression, but not PKCδ, by siRNA inhibited Rac and PAK activity as well as invasiveness. Loss of PKCα restored focal adhesion formation and partially restored stress fiber formation, while loss of PKCδ primarily restored stress fibers.

**Conclusion:**

The misregulated expression of PKCα and PKCδ and elevated Src activity in metastatic melanoma cells is required for efficient αvβ3-mediated invasion. PKCα and Src enhance αvβ3-mediated invasion in part by increasing the GTPase activity of Rac relative to RhoA. PKCα influences focal adhesion formation, while PKCδ controls stress fibers.

## Background

The development of metastatic melanoma in human patients is marked by the progression from a noninvasive radial growth phase to a vertical growth phase, where tumor cells begin to penetrate through the dermal layer and into the subcutaneous tissues [1]. Induced expression of αvβ3 integrin is coincident with and present only on vertical growth phase melanomas [2,3]. αvβ3 is not expressed on melanocytes, nevi, or radial growth primary melanomas *in vivo*. It is present in melanocyte precursors, i.e. neural crest cells, as they migrate and populate the skin during early development. The M21-L human melanoma cell line, lacking αvβ3 integrin expression, was shown to have a dramatically reduced ability to induce tumors and metastases in nude mice. Expression of αvβ3 in M21-L cells restored their tumorigenic properties [4]. Furthermore, subcutaneous growth of αvβ3-positive M21 melanoma cells in nude mice was reduced significantly by αv-specific blocking antibodies. Ectopic expression of αvβ3 in radial growth melanomas isolated from patients is sufficient to confer enhanced growth and invasive properties to those tumor cells [5]. Thus αvβ3 integrin plays an important role in both tumor growth and metastasis. However, the precise mechanisms by which αvβ3 integrin expression promotes melanoma growth and metastasis remain poorly understood.

αvβ3 integrin functions as an adhesion receptor on the surface of cells and binds with highest affinity to the extracellular matrix (ECM) ligand vitronectin (VN). Binding is mediated via RGD peptides present in VN. RGD peptides are also present in other ECM proteins, such as fibronectin and fibrinogen, and correspondingly αvβ3 is able to mediate adhesion to these ECM proteins as well. Engagement of αvβ3 integrin upon adhesion to matrix, like the engagement of most integrins by the ECM, triggers intracellular signaling [6]. The initial attachment of cells to matrix induces the formation of early integrin focal contact structures or nascent adhesions that require signaling from Src and include the integrin binding protein talin, ERM proteins, cortactin, and other signaling molecules such as PKC and the Rho GTPase Rac [7,8]. With time the focal complexes mature into integrin-rich focal adhesions containing structural proteins such as vinculin, paxillin, α-actinin, and FAK, followed by the emergence of bundled actin stress fibers which project from the focal adhesions. The maintenance of these structures is mediated by signaling to the small GTPase RhoA [9]. For matrix adherent cells to migrate they must disrupt their focal adhesions and actin stress fibers, and loosen their adhesion to the ECM [10]. As the cell reattaches its loosened membrane the early focal contact structures are again reformed. A hallmark of highly migratory cells is diminished focal adhesions and stress fibers and the predominance of the early focal contact structures. Maintenance of these structures is regulated by cdc42 and Rac [9].

Formation of the different integrin adhesion structures is tightly controlled by signaling molecules that target the structural proteins involved in their development. The nonreceptor tyrosine kinase Src has been shown to physically interact with the cytoplasmic domain of the β3 integrin tail and is activated immediately upon integrin engagement [11]. Loss of Src kinases dramatically impairs integrin-mediated focal contact assembly and over expression of active Src prevents focal adhesion and stress fiber formation [12]. Structural and regulatory proteins within focal contacts and focal adhesions, such as cortactin, ERMs, Rho GEFs, FAK, paxillin, vinculin, and Rho GAPs, are known Src kinase substrates. Thus Src plays a pivotal role in integrin-mediated adhesion and signaling. A few reports have linked increased Src kinase signaling and expression with the development of metastatic melanoma. Elevated Src activity, over expressed Yes, and active Fyn have all been reported in melanoma cell lines [13-15]. Src kinase targets identified in melanoma cells include cortactin, tenascin-C, Tks5, and Stats [15-18]. Increased Src signaling in melanoma cells was associated with increased growth, survival, and metastasis and in two cases increased Src signaling was linked to αvβ3 integrin [17,19].

The most well characterized substrate of the serine/threonine kinase PKC, MARCKS, is an actin binding protein localized within cortical actin structures at the cell membrane [20]. Adhesion to matrix results in PKC-dependent phosphorylation and removal of MARCKS from the actin structures. Displacement of MARCKS is thought to be important for reorganization of the cortical actin required for the assembly of focal contacts. Changes in PKC expression have been implicated in melanoma progression. PKCα was shown to be highly expressed in some metastatic melanomas and was crucial for melanoma metastasis *in vivo *[21,22]. However, some melanoma cell lines do not express PKCα due to a deletion in the gene [23]. PKCβ, which is involved in maintaining melanocyte differentiation, is often lost in metastatic melanoma [24]javascript:PopUpMenu2_Set(Menu7678596);. On the other hand over expression of PKCδ, in at least one melanoma cell line, correlated with increased metastasis [25]. The extent to which αvβ3 integrin in particular activates or cooperates with PKC to regulate melanoma metastasis has not been investigated and which PKC isoform may be responsible for αvβ3-dependent melanoma invasion and metastasis has not been determined.

While screening the expression levels of PKC and Src in various melanoma cell lines we discovered a correlation between high PKCα and PKCδ levels and Src activity, and the metastatic potential of several melanoma cell lines. In the study presented here we tested the hypothesis that elevated PKC levels and Src kinase activity are responsible for αvβ3-dependent invasion of highly metastatic melanoma cells.

## Methods

### Antibodies

αvβ3 (LM609) and β5 (P1F6) blocking integrin antibodies [26,27] were purchased from Chemicon. The Src 327 monoclonal antibody has been described previously [28]. Src phospho-specific antibody, Y418, was purchased from Biosource. Antibodies to PKCα, PKCδ, PKCε, Rac1, and RhoA were obtained from BD Transduction Labs. PAK and phospho-PAK antibodies were purchased from Cell Signaling Technology. p190RhoGAP antibodies came from Santa Cruz. Alexafluor 488 goat anti-mouse and Alexafluor 546 phalloidin were from Molecular Probes and Hoechst 33258 was from Sigma.

### Cell lines

The metastatic melanoma cell line C8161.9 [29], obtained from Dr. Danny Welch, (University of Alabama, Birmingham, Alabama, USA) was grown in DMEM/F12 (Invitrogen) supplemented with 2 mM L-glutamine, 50 U/ml penicillin, 50 ug/ml streptomycin/ml, 0.1 mM NEAA, 1 mM NaPyruvate, and 5% fetal bovine serum (FBS). M14 melanoma cells (Dr. Han-Mo Koo, Van Andel Institute) were grown in RPMI-1640 (Invitrogen) supplemented with 2 mM L-Glutamine, Gentamicin, and 5% FBS. A375 metastatic melanoma cells were obtained from ATCC and grown in MEM Hanks (Invitrogen) supplemented with 50 U of penicillin, 50 ug/ml streptomycin, 0.1 mM NEAA, 1 mM NaPyruvate, and 10% FBS. HUVECS were obtained from Cascade Biololgicals and normal cultured primary melanocytes, NHEM, were purchased either from Clonetics or Cascade. Both cell lines were grown in media supplied by their respective companies. DF1 chicken fibroblast cell line [30] (Dr. Bart Williams, Van Andel Institute) was grown in DMEM (Invitrogen) supplemented with 2 mM L-Glutamine, 1 mM NaPyruvate, 50 U/ml penicillin, 50 ug/ml streptomycin, and 10% heat inactivated FBS. Stable C8161.9 and M14 cell lines expressing tVA, the chicken retrovirus receptor, were established as previously described [31].

### Inhibitors

Src inhibitors PD173955, PD180970, and SU6656, and PKC inhibitors bisindolylmaleimide and 12-O-tetradecanoylphorbol 13-acetate (TPA) were purchased from Calbiochem. Cells were treated overnight with 100 ng/ml TPA or for 30 minutes prior to use with 10 uM bisindolylmaleimide, 10 uM PD173955 or PD180970, or 2 uM SU6656.

### Transient Transfections

One ug of pcDNA-RhoA-Q61L [32] was transiently cotransfected with 1 ug pCMV-GFP into C8161.9 cells with Lipofectamine Plus (Invitrogen) using the recommended protocol. 48 hours after transfection cells were plated on matrix for immunofluorescent staining.

### Virus Generation and Infections

pGST-Src and pGST-SrcK295M [33] were obtained from Dr. Sara Courtneidge (Burnham Institute, San Diego, California, USA) and subcloned via EcoRI into pENTR3c vector (Gateway, Invitrogen) and then recombined into the Gateway modified pDEST-RCAS retroviral vector [34]. Full length rabbit PKCα and mouse PKCδ cDNAs from Dr. Shigeo Ohno (Yokohama City University, Japan) were subcloned via EcoRI into Bluescript cloning vector (Strategene) to generate pBS-PKCα and pBS-PKCδ. PKCα and PKCδ were released with NotI and ClaI and subcloned into pRCASBP-Y retroviral vector [34]. RCAS-Src, RCAS-SrcK295M, RCAS-PKCα, and RCAS-PKCδ were transfected into DF1 chicken fibroblasts and retroviruses generated as previously described [30]. C8161.9 cells or M14 cells expressing tVA were infected with virus (~1 × 10^7 ^IU/100 mm plate) in the presence of 8 ug/ml Polybrene (Invitrogen). Seventy-two hours after infection, cells were selected in 7 ug/ml of puromycin (Invitrogen) or 400 ug/ml G418 (Invitrogen) for 2 weeks. Surviving cells were pooled and maintained in standard DMEM/F12 growth medium.

### siRNAs

PKC isoform-specific siRNAs sequences (IDT) were as follows: PKCδ si1δ: 5'-UGGCGCCGUUCCUGCGCAU-3', PKCα si2α: 5'-CCUGCGAUAUGAACGUUCA-3', and si3α: 5'-AAGGCUUCCAGUGCCAAGA-3'. The scrambled siRNA control sequence was 5'-ACUACCGUUGUUAUAGGUG-3'. All sequences were blasted using BLASTN and contained no homology to any other known human genes. C8161.9 cells at 30–50% confluency in 100 mm tissue culture dishes were starved (DMEM/F12 + 0.1% FBS) 24 hours prior to transfection. Cells were transfected with 20 nM siRNAs with SiLentFect (Bio-Rad) as suggested by the manufacture. The cells were allowed to recover in growth media supplemented with 2% FBS for 24 hours. Cells were then placed in starvation media for an additional 48 hours before use in adhesion assays.

### FACS

Serum starved cells were removed from tissue culture plates by trypsinization and rinsed with soybean trypsin inhibitor (Invitrogen). Cells resuspended in PBS + 0.1% BSA at 1 × 10^6  ^to 1 ×10^7 ^cells/ml were incubated with 5 ug/ml of αvβ3 or β5 integrin antibodies for 1 hour at 4°C. Cells were washed with PBS and incubated with FITC-conjugated secondary antibodies in PBS+0.1% BSA for 1 hour at 4°C. Cells were washed with PBS, fixed with 1% paraformaldehyde in PBS at 4°C for 20 minutes, washed in PBS, and analyzed by Fluorescent Activated Cell Sorting (FACS) with the FACSCalibur (Becton Dickinson) and CellQuest (Becton Dickinson).

### Matrix Adhesion

For assays done on extracellular matrices, cells were prepared as previously described [35]. Briefly, cells were growth factor-starved for 48 hours, trypsinized, treated with soybean trypsin inhibitor (Invitrogen), washed in PBS, and suspended in serum-free medium for 30–60 minutes. Cells were then plated on tissue culture plates or sterilized coverslips coated with 5 ug to 10 ug/ml vitronectin (Chemicon) and blocked with 1% BSA (Sigma). Suspension controls were maintained at 37°C. One to two hours after plating on matrix cells were either lysed for biochemical analysis or immunostained.

### Immunoblotting

For biochemical analyses cells were lysed either in Triton-X (50 mM Tris pH 7.5, 100 mM NaCl, 0.5 mM EDTA, 1% TritonX-100, 50 mM NaF, 50 mM β-glycerophosphate, 5 mM sodium pyrophosphate, 1 mMNa_3_VO_4_, 1 mM PMSF, 100 U/ml aprotinin, 10 ug/ml pepstatin, and 10 ug/ml leupeptin) or RIPA (10 mM Tris pH 7.2, 158 mM NaCl, 1 mM EDTA, 0.1% SDS, 1% NaDOC, 1% Triton-X100, 1 mMNa_3_VO_4_, 1 mM PMSF, 100 U/ml aprotinin, 10 ug/ml pepstatin, and 10 ug/ml leupeptin) buffers. For immunoprecipitation, 500 to 1000 ug/ml protein was incubated with the appropriate antibodies for 3 hours at 4°C with either protein A- or protein G-conjugated agarose beads (Pierce) to capture the complexes. Washed immunoprecipitates were resuspended in 2× SDS sample buffer containing 10% β-mercaptoethanol. All samples were subjected to SDS-polyacrylamide gel electrophoresis, and transferred to a polyvinylidene difluoride membrane (PVDF). The PVDF membranes were blocked with 5% BSA in Tris-buffered saline containing 0.1% Tween 20 (TBST) for 2 hours, followed by 2 hour incubation with the appropriate primary antibodies in 5% BSA/TBST. After washing, blots were incubated with a horseradish peroxidase-conjugated secondary antibody for 1 hour in 5% BSA/TBST, visualized with a chemiluminescence reagent, and captured by a CCD camera in a Bio-Rad Chemi-Doc Imaging System and quantified using Quantity One software (Bio-Rad). Blots were stripped in low-pH 2% SDS at 65°C for 60 minutes, rinsed and reprobed for total levels of protein in the immunoprecipitates or cell lysates.

### Migration and Invasion assays

Boyden chambers inserts containing Matrigel in the upper chamber were purchased from Becton-Dickinson. The lower side of the porous membrane in the Boyden inserts was treated with 10 ug/mL vitronectin overnight at room temperature. This results in a vitronectin coating on the lower side of the membrane as well as some vitronectin seeping into the Matrigel above. After coating, the lower side of the membranes were washed with PBS and blocked with 1% BSA for 2–4 hours at 37°C while the Matrigel in the upper chamber was rehydrated with DMEM. Serum starved cells were placed in suspension in serum free medium. Inhibitors or blocking antibodies were added to suspension cells prior to plating in chambers and 1.5 × 10^5 ^to 2 × 10^5 ^cells were added to the top chamber in the absence of serum. Medium without serum was added to the lower chambers. All chambers were incubated in 5% CO_2 _at 37°C for 72 hours. Chamber inserts were rinsed with PBS and then cells were stained and fixed with crystal violet/formalin mixture (Chemicon) for 20 minutes. Any cells remaining in the top chamber were removed with a cotton applicator tip. Cells having migrated to the bottom chamber and adhering to the lower side of the membrane were visualized with Nikon Eclipse TE300 Epi-flourescence Microscope using a 10× objective. Pictures were taken with the Hamamatzu Digital CCD camera using OpenLab imaging software (Improvision) and the RGB Colour Merge Automator (OpenLab). Five pictures of each well were taken and the cells in each image were counted. All assays were repeated at least 3 times.

### Rac and Rho activation

Rho pull-down assays were carried out essentially as described [36] and the Rac pull-down protocol was adapted from del Pozo et al [37]. GST-RBD (rhotekin binding domain) and GST-PBD (PAK binding domain) protein expression was induced with 0.5 mM Isopropyl-β-D-thiogalactopyranoside (IPTG) for 2 hr at 30°C in 4 liters of culture or for 3–4 hr at 37°C in 200 ml respectively. GST-RBD bacteria were resuspended in 40 ml cold lysis buffer (50 mM Tris, pH 7.5, 1% Triton X-100, 150 mM NaCl, 5 mM MgCl_2_, 1 mM DTT, 10 ug/ml aprotinin, 10 μg/ml leupeptin and 1 mM PMSF). GST-PBD bacteria were resuspended in 10 ml cold lysis buffer (50 mM Tris, pH 7.5, 150 mM NaCl, 10% glycerol, 2 mM EDTA, 10 ug/ml aprotinin, 10 ug/ml leupeptin and 1 mM PMSF). Suspensions were sonicated and clarified lysates were mixed with 0.6 ml glutathione beads (Sigma) and rotated at 4°C for 60 min. The GST-RBD beads were washed six times with 12 ml wash buffer (50 mM Tris, pH 7.5, 0.5% Triton X-100, 150 mM NaCl, 5 mM MgCl_2_, 1 mM DTT, 1 ug/ml aprotinin, 1 ug/ml leupeptin and 0.1 mM PMSF) and GST-PBD beads were washed three times with 12 ml lysis buffer supplemented with 0.5% NP-40 then washed 3× with storage buffer (50 mM Tris pH7.5, 150 mM NaCl, 10% glycerol, 5 mM MgCl_2_, 1 mM DTT). Beads were resuspended in 1 ml wash buffer with 10% glycerol and used immediately for pull-downs. Purified GST-RBD and GST-PBD proteins were quantified using a 12% SDS PAGE and BSA standards for comparison. These preparations typically yielded between 20–30 ug protein per 20 ul aliquot of bead suspension.

Following adhesion to matrix cells were placed on ice and washed twice with ice-cold Tris-buffered saline (50 mM Tris, pH7.4, 150 mM NaCl, 10 mM MgCl_2_). For Rho assays cells were lysed in 500 ul of cold Rho lysis buffer (50 mM Tris, pH 7.2, 1% Triton X-100, 0.5% sodium deoxycholate, 0.1% SDS, 500 mM NaCl, 10 mM MgCl_2_, 10 μg/ml each of leupeptin and aprotinin, and 1 mM PMSF). For Rac assays, cells were lysed in 500 ul of cold Rac lysis buffer (50 mM Tris pH 7.4, 10 mM MgCl_2_, 1% Triton-X, 10% glycerol, 500 mM NaCl, 0.1% DOC, 10 ug/ml leupeptin, 10 ug/ml aprotinin, 10 ug/ml pepstatin, 2 mM PMSF). 300–500 ug of lysates were combined with 20 ul of bead suspension and rotated at 4°C for 60 min. Bead suspension was washed four times with 600 μl ice cold buffer containing 50 mM Tris, pH 7.2, 1% Triton X-100, 150 mM NaCl, 10 mM MgCl_2_, and 10 ug/ml each of leupeptin and aprotinin, and 0.1 mM PMSF. Samples were boiled 10 min in SDS sample buffer and separated on 12% Tris-Glycine SDS PAGE gels. Proteins were transferred to PVDF membrane for immunoblotting with RhoA or Rac antibodies.

ECL images were captured by a CCD camera in a Bio-Rad Chemi-Doc Imaging System and the intensity of the activated RhoA and total RhoA ECL signals were quantified using Quantity One software (Bio-Rad). Extent of RhoA activation was normalized to total RhoA for each sample. Data were expressed as the fold in increase in RhoA activation compared to control or untreated samples.

### Immunofluorescent staining

Sterilized cover-slips (Fisher) were coated with 5–10 μg/ml vitronectin O/N at 4°C, blocked in PBS + 1% BSA for 1–2 hours at 37°C, and then washed with PBS prior to plating 2–3 × 10^5 ^suspended cells in serum-free medium. After incubation at 37°C for 45 minutes cells were fixed with 4% paraformaldehyde in PBS at 4°C for 20 minutes. Cells were permeabilized with TBS (10 mM Tris, pH 8.0, 150 mM NaCl) + 0.5% TritonX-100 for 10 minutes at room temperature and blocked with 2% BSA/TBS-T for 20 minutes at room temperature. Cells were then incubated at room temperature with primary antibody and Phalloidin 546 in 2% BSA/TBS-T for 60 minutes and then incubated with Alexaflour 488 goat anti-mouse in 2% BSA in TBS-T for 60 minutes. Nuclei were stained with Hoechst for 5 minutes at room temperature. Cover-slips were mounted onto slides in 50% glycerol. Cells were visualized with Nikon Eclipse TE300 Epi-flourescence Microscope using a 60× oil immersion objective. Pictures were taken with the Hamamatzu Digital CCD camera using OpenLab imaging software (Improvision).

## Results

### Vitronectin integrin expression in metastatic melanoma cells

**Figure 1:**
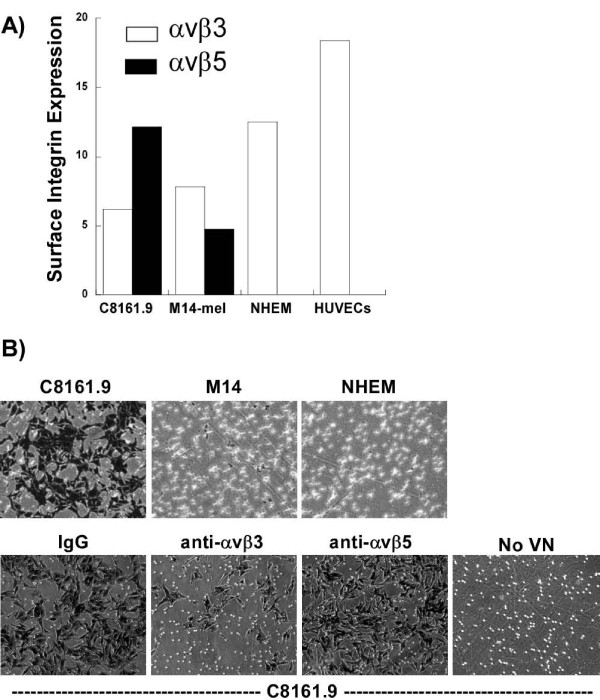
**αvβ3 integrin-dependent invasion of C8161.9 cells**. A) Cell surface expression levels of αvβ3 (clear bar) and αvβ5 (black bar) integrins in two metastatic melanoma cell lines, C8161.9 and M14-mel, normal cultured melanocytes (NHEM), and the endothelial cell line HUVEC, were measured using FACS after surface labeling with fluorescently-labeled anti-integrin antibodies. B) Vitronectin-induced Matrigel invasion of C8161.9, M14, or normal cultured melanocytes (NHEM) in Boyden chambers in the absence of serum or growth factors was assessed in the absence (IgG) or presence of anti-integrin blocking antibodies (anti-αvβ3 or anti-αvβ5). In the absence of vitronectin (no VN), cells were unable to invade the Matrigel.

The primary ligand for αvβ3 is vitronectin (VN); however, another integrin, αvβ5, also binds VN. To determine which integrin is responsible for the biological effects mediated by adhesion to VN, αvβ3 and αvβ5 integrin expression was measured in the melanoma cells. The cell surface levels of αvβ3 and αvβ5 in cultured melanocytes and in a non-invasive cell line, M14 [38], and a highly metastatic melanoma cell line, C8161.9 [29], were quantified by FACS. Irrespective of their metastatic potential, both melanoma cell lines expressed similar levels of αvβ3, while normal cultured melanocytes and HUVECs (positive control) expressed over 2 times more αvβ3. On the other hand, the more highly metastatic melanoma cell line, C8161.9, expressed over 2× higher levels of αvβ5 than M14 (Figure 1A). To determine which integrin is required for the migratory and invasive properties of the melanoma cell lines, cells were treated with or without VN integrin-blocking antibodies [26,27] or IgG and their ability to invade and migrate through vitronectin-enriched Matrigel to the lower chamber of a Boyden chamber in the absence of serum or growth factors was evaluated. M14 and normal melanocytes both failed to invade through VN/Matrigel, while invasion of the highly metastatic C8161.9 cells was dramatically diminished in the presence of αvβ3, but not αvβ5 blocking antibodies (Figure 1B). If VN is omitted C8161.9 cells are unable to invade. Thus, expression of αvβ3 is required for invasion of highly metastatic melanoma cells. However, αvβ3 expression alone is not sufficient, since αvβ3 is expressed on both M14 and normal cultured melanocytes and these cells were unable to invade. Therefore, additional signaling events must also be required. These signaling events must be intrinsic to the cells, since no exogenous growth factors, serum, or additional external stimuli other than extracellular matrix (ECM) proteins, were added to the invasion assays.

### Src regulates invasion of metastatic melanoma cells

**Figure 2:**
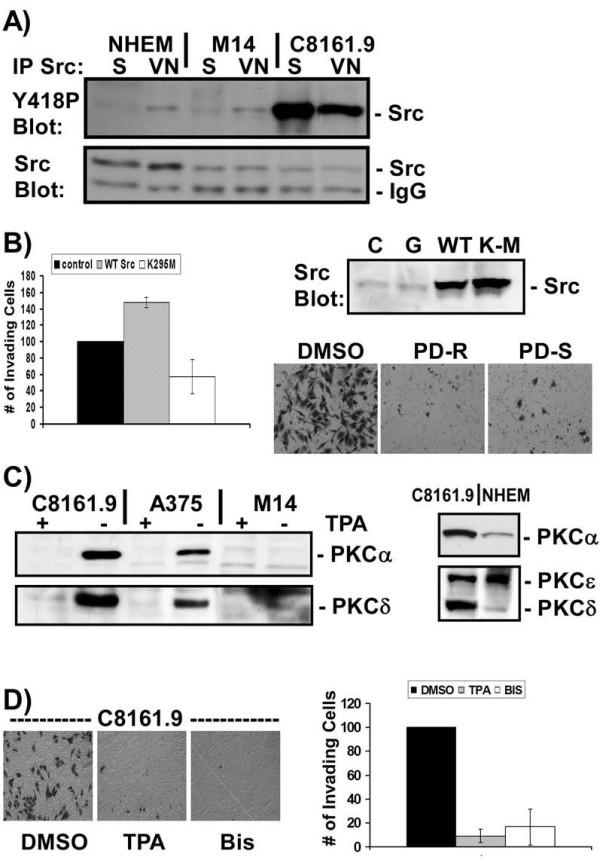
**Src and PKC are required for αvβ3-dependent invasion**. A) C8161.9, M14, or normal cultured melanocytes (NHEM) were placed in suspension (S) or plated on vitronectin (VN). The level of Src activity present in Src immunoprecipitates was measured by immunoblotting with rabbit anti-phospho-specific antibodies to the activation loop phosphorylated tyrosine, Y418 (Y418P Blot). Total levels of Src in the same immunoprecipitates were measured by immunoblotting stripped blots with total mouse anti-Src antibodies (Src Blot). The mouse immunoglobulin band in the immunoprecipitates is indicated (IgG). B) C8161.9 cells expressing the avian retroviral receptor tVA were left uninfected (C), or infected with avian retroviruses expressing GFP (G), wild type Src (WT) or a dominant interfering Src mutant (K-M or K295M). Levels of Src expression were monitored by immunoblotting of whole cell lystates (Src). Vitronectin-induced Matrigel invasion of Src-inhibited cells was quantified. Data represents 6 independent experiments. C8161.9 cells were also treated with two Src inhibitors, PD173955 (PD-R) or PD180970 (PD-S) and the extent of VN-induced invasion in the absence of serum or growth factors was assessed. C) Three melanoma cells lines, C8161.9, A375, M14, and normal cultured melanocytes (NHEM) were untreated (-) or pretreated (+) with 100 nM TPA for 16 hours to down regulate expression of classical and novel PKCs. The levels of PKCα, PKCδ, and PKCε expression were measured by immunoblotting of whole cell extracts. Equal levels of cell extract were loaded and probed by immunoblotting with specific antibodies. D) C8161.9 cells were pretreated with DMSO, TPA for 16 hours, or bisindolylmaleimide and vitronectin-induced Matrigel invasion in the absence of serum or growth factors was assessed and quantified.

αvβ3 integrin is a strong activator of the nonreceptor tyrosine kinase Src [11], which is known to regulate cell migration and invasion [39]. Therefore, to determine whether Src is required for the invasive properties of C8161.9 cells, the levels of Src protein and its activity were first measured by immunoblotting of cell extracts from normal melanocytes, M14, and C8161.9 cells after adhesion to VN. There were no significant differences in total levels of Src protein between these cell lines; however, the level of activated Src (as measured by phosphorylation at Y418) was dramatically increased in the highly metastatic C8161.9 cells (Figure 2A). A similar decrease in phosphorylation at Y529 was also observed (data not shown). Strikingly this increase in Src activity occurred independently of integrin engagement, as Src activity was high even in cells placed in suspension. Next, Src activity was inhibited in C8161.9 cells and αvβ3-dependent invasion was measured. Over expression of a dominant interfering form of Src (SrcK295M) [33] or treatment with two Src inhibitors dramatically reduced C8161.9 cell invasion (Figure 2B). Over expression of wild type Src caused a 50% increase in invasion. Normal cultured melanocytes and M14, both express αvβ3 integrin (Figure 1A); however, neither have high levels of active Src. Thus, constitutive activation of Src, in combination withαvβ3, is required for invasion by highly metastatic melanoma cells.

### PKC regulates invasion of metastatic melanoma cells

Elevated expression of PKC has been shown to be associated with metastatic melanoma [21,22]. Highly metastatic melanoma cells may also require PKC for αvβ3-mediated invasion. The two highly invasive cell lines, C8161.9 and A375, expressed high levels of both PKC*α *and PKCδ compared to the less invasive melanoma cell line M14 and normal cultured melanocytes (Figure 2C). PKC activity was inhibited either by treatment with the PKC-specific inhibitor bisindolylmaleimide, or down regulation of PKC expression by long term treatment with TPA. Under these conditions, αvβ3-mediated invasion of C8161.9 cells was inhibited 5–10 fold (Figure 2D). Thus, PKC activity is required for invasion of C8161.9 cells.

### Focal adhesion and stress fiber formation is aberrant in metastatic melanoma cells

**Figure 3:**
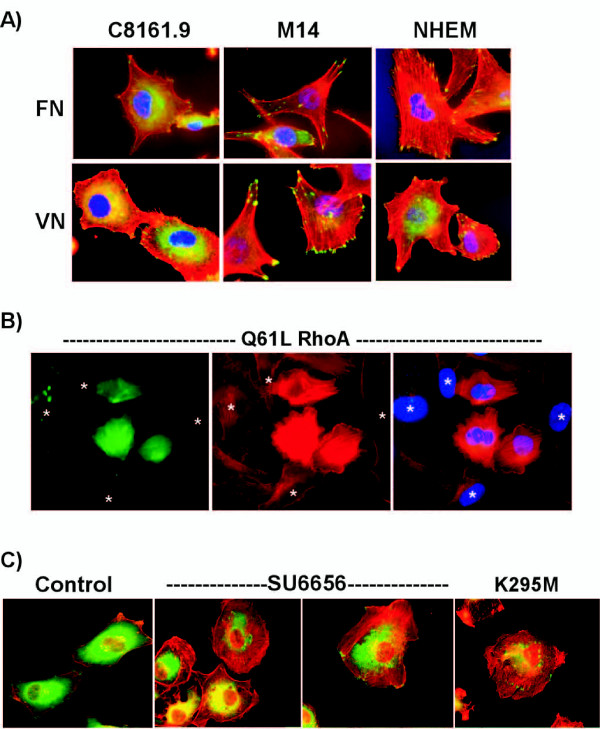
**Src activation prevents RhoA-dependent formation of stress fibers and focal adhesions**. A) C8161.9, M14, or normal cultured melanocytes (NHEM) were plated on fibronectin (FN) or vitronectin (VN) for 2 hours and the extent of stress fiber formation was monitored by staining with phalloidin (red). Focal adhesion formation was monitored by immunostaining with paxillin antibodies (green). Cells were counterstained with Hoescht to detect nuclei (blue). B) C8161.9 cells were transiently transfected with GFP expressing plasmid and an active form of RhoA (Q61L). Cells were plated on vitronectin for 2 hours and stained with phalloidin (red) to monitor actin structures in GFP-positive transfected (green) cells. Only the three cells expressing green GFP (i.e. transfected with RhoA (Q61L)) displayed prominent stress fibers. The nuclei of cells not expressing GFP/RhoA Q61L are marked by asterisks. C) C1861.9 cells were left untreated (control), treated with SU6656, or infected with dominant interfering mutant Src (K295M) virus. Cells were plated on vitronectin for 2 hours and stress fiber formation monitored by staining with phalloidin (red) and focal adhesion formation by immunostaining with paxillin antibodies (green).

Adhesion of normal cultured melanocytes or M14 melanoma cells to VN induced the formation of multiple stress fibers and large focal adhesion complexes from which the stress fibers emanated (Figure 3A). However, adhesion of C8161.9 cells to VN failed to induce stress fiber formation and the number and size of focal adhesions were dramatically reduced. Since RhoA has been shown to regulate the formation of stress fibers and focal adhesions, RhoA signaling may be impaired in C8161.9 cells. To test this C8161.9 cells were transiently cotransfected with a constitutively active form of RhoA and GFP. Only the three cells expressing green GFP (i.e. transfected with and expressing constitutively active RhoA Q61L) displayed prominent stress fibers (Figure 3B). The surrounding cells (asterisks) not expressing GFP and RhoA Q61L do not have prominent stress fibers. Src has been shown to be a negative regulator of RhoA activity [40]. Therefore, it is possible that the high levels of Src activity in C8161.9 cells may be inhibiting RhoA activity. Indeed, inhibition of Src with the specific inhibitor SU6656 or over expression of the K295M Src dominant interfering mutant was sufficient to induce stress fibers and increase focal adhesion formation (Figure 3C). Thus, the aberrant stress fiber and focal adhesion formation in C8161.9 cells plated on VN is likely due to the constitutively high level of active Src, which effectively down regulates RhoA activity.

### Src negatively regulates RhoA activity in metastatic melanomacells

**Figure 4:**
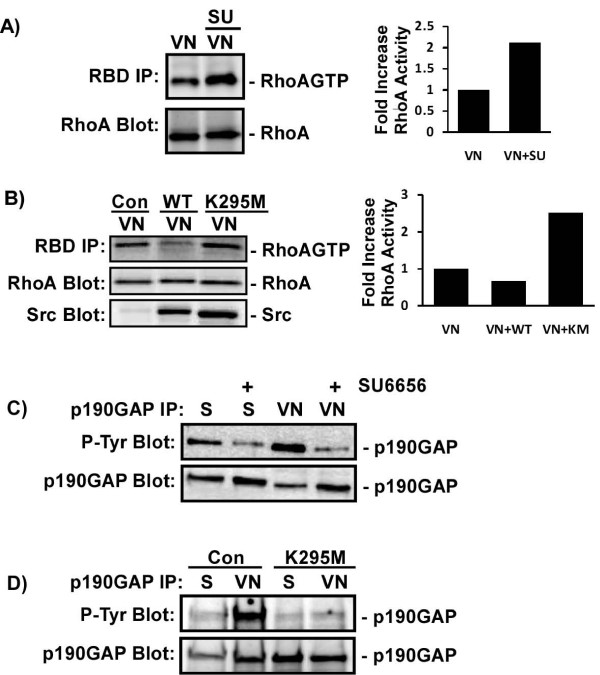
**Src negatively regulates RhoA activity in C8161.9 cells**. A, C) C8161.9 cells were left untreated, treated with SU6656 (SU), or B, D) infected with no virus (control), wild type Src (WT Src), or dominant interfering mutant Src (SrcK295M) virus. Cells were then placed in suspension (S) or plated on vitronectin (VN) for 45 minutes. A, B) the amount of GTP-bound RhoA (RhoAGTP) was monitored using GST-RBD pull-down assays (RBD IP) and immunoblotting. Total levels of RhoA and over expressed Src in the cell extracts were monitored by immunoblotting (RhoA Blot, Src Blot). Extent of RhoA activation was normalized to total RhoA for each sample. Data were expressed as the fold in increase in RhoA activation compared to control or untreated samples. C, D) The level of p190RhoGAP tyrosine phosphorylation was measured by immunoblotting of immunoprecipitates (p190GAP IP) with anti-phosphotyrosine antibodies (P-Tyr Blot). Total levels of p190RhoGAP in the immunoprecipitates were measured by immunoblotting with anti-p190RhoGAP antibodies (p190GAP Blot).

To further verify that Src is regulating RhoA activity, RhoA activity was measured in C8161.9 cells plated on VN using GST-RBD pull-down assays. Inhibition of Src activity with the Src-specific inhibitor SU6656 resulted in a 2.1-fold increase in RhoA activation after plating on VN (Figure 4A). Similarly, expression of the dominant interfering mutant of Src (SrcK295M) increased RhoA activity 2.5-fold compared to control cells plated on VN (Figure 4B). Conversely, over expression of wild type Src decreased RhoA activation 1.4-fold. The Rho-specific GAP, p190RhoGAP, is known to be activated by adhesion to matrix and its activity is regulated by Src-dependent tyrosine phosphorylation [41-43]. Activation of p190RhoGAP by active Src in C8161.9 cells could be responsible for the inhibition of RhoA activity following adhesion to matrix. Treatment of C8161.9 cells with the Src inhibitor SU6656 or expression of mutant Src blocked VN-induced tyrosine phosphorylation of p190RhoGAP (Figure 4C, D). All together these data demonstrate that αvβ3-mediated adhesion of highly metastatic melanoma cells to its extracellular matrix substrate VN results in Src-dependent phosphorylation and up-regulation of p190RhoGAP activity, a Src-dependent decrease in RhoA activity and stress fiber and focal adhesion formation, and a Src-dependent increase in αvβ3-mediated invasion. Thus, the elevated Src activity in the highly invasive metastatic melanoma cells is required for effective αvβ3-mediated invasion and functions in part to limit RhoA activity, which may favor a more migratory phenotype.

### PKCα positively regulates Rac activity in metastaticmelanoma cells

**Figure 5:**
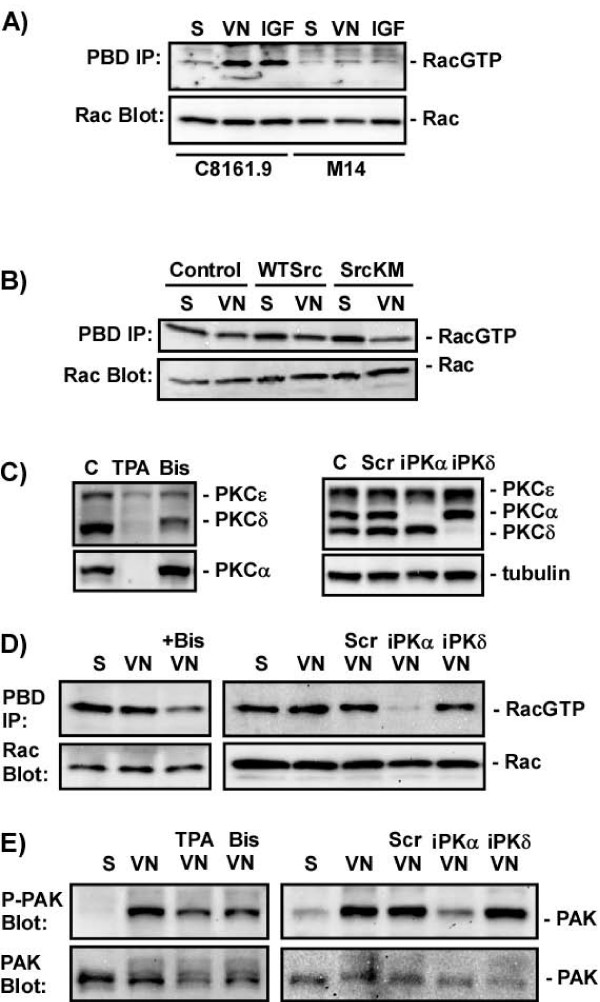
**PKCα positively regulates Rac activity in C1861.9 cells**. A) C8161.9 or M14 cells were placed in suspension (S), plated on vitronectin (VN) for 45 minutes, or confluent cells were serum starved and then stimulated with 40 uM IGF (IGF) for 5 minutes. The level of GTP-bound Rac (RacGTP) was measured using GST-PBD pull-down assays (PBD IP) and immunoblotting for Rac activity. Total levels of Rac were monitored by immunoblotting of whole cell extracts (Rac Blot). B) C8161.9 cells uninfected (control) or infected with wild type (WT Src) or dominant interfering Src mutant (SrcKM) virus were treated and plated as in A) and analyzed for Rac activation (PBD IP). C) C8161.9 cells were pretreated with DMSO, TPA for 16 hours, or bisindolylmaleimide (Bis). Or cells were transfected with nothing (C), scrambled (Scr), PKCα (iPKα), or PKCδ (iPKδ) siRNA and the total levels of PKCα, PKCδ, and PKCε were monitored by immunoblotting. Total levels of protein were monitored by immunoblotting for tubulin. D) C8161.9 cells were treated with DMSO or bisindolylmaleimide, or transfected with scrambled (Scr), PKCα (iPKα), or PKCδ (iPKδ) siRNA and their ability to activate Rac (PBD IP) in suspension (S) or after plating on vitronectin (VN) was assessed as in A). E) Cells were treated as in D) and PAK activation was measured by immunoblotting of whole cell lysates with anti-phospho-PAK antibody that recognizes the activated form of PAK (P-PAK Blot). Total levels of PAK in the cell lysates were monitored by immunoblotting with anti-PAK antibodies (PAK Blot).

Previous studies have demonstrated that the relative differences in RhoA and Rac activity in cells reflects the net migratory phenotype [44]. Cells with high Rac, but low RhoA, are more migratory, while cells with high RhoA, but low Rac, are less migratory. Given that C8161.9 cells have low RhoA activity and their adhesion and actin structures resemble those seen in cells with high Rac activity, elevated Rac activity may account in part for the increased invasiveness of C8161.9 cells. The total levels of Rac protein and Rac activity were measured and compared between C8161.9 and M14 cells. Using cell lysates containing comparable levels of Rac expression and normalizing for total Rac levels, 2.5–3-fold more Rac activity is present in C8161.9 cells than M14 cells (Figure 5A).

One possibility is that the elevated Src activity in C8161.9 cells not only inhibits RhoA, but also increases Rac activity. To test this hypothesis, Src activity was inhibited in C8161.9 cells and the effects on Rac activity monitored. Surprisingly, inhibition of Src activity with the dominant interfering Src mutant (Figure 5B) or treatment with Src inhibitors (not shown) had no effect on Rac activity. Downstream signaling to the Rac effector PAK was similarly unaffected by Src inhibition (data not shown). Thus the elevated Rac activity in C8161.9 cells is not due to elevated Src activity.

PKC activity was inhibited in C8161.9 cells, either by long term treatment with TPA to inhibit PKC expression (Figure 5C) or with the catalytic inhibitor bisindolylmaleimide, and the effect on Rac activation after plating on VN was analyzed. Inhibition of PKC resulted in reduced Rac activity (Figure 5D) and reduced activation of the Rac effector PAK (Figure 5E). To determine which PKC isoform, PKCα or PKCδ, was responsible for regulating Rac activity an siRNA approach was used. Transient transfection of siRNAs specific to PKCα or PKCδ specifically inhibited their respective expression, while the scrambled siRNA had no effect on either PKC isoform (Figure 5C). Inhibition of expression was maximal at 72 hours and remained sustained for up to at least 144 hours (data not shown). None of the siRNAs had any effect on PKCε. Inhibition of PKCα, but not PKCδ, expression in C8161.9 cells by PKC siRNAs dramatically reduced Rac activity (Figure 5D). Loss of PKCα, but not PKCδ, also reduced VN-induced PAK activity (Figure 5E). Thus, PKCα is required for activation of Rac and its downstream effector PAK in C8161.9 cells.

Interestingly, Rac activity was often elevated in suspension cells (Figure 5B, D), while PAK activity was not (Figure 5E) suggesting an apparent disconnect between these two molecules. However, previous studies have demonstrated that the ability of Rac to signal to PAK is dependent on cell adhesion [37]. The ability of Rac to activate PAK is dependent on membrane translocation of Rac, which requires an integrin-dependent adhesion event. Thus, even though Rac is sometimes highly active in suspended C8161.9 cells, it cannot complete the circuit to signal to PAK in the absence of integrin engagement.

### Both PKCα and PKCδ contribute to invasion

**Figure 6:**
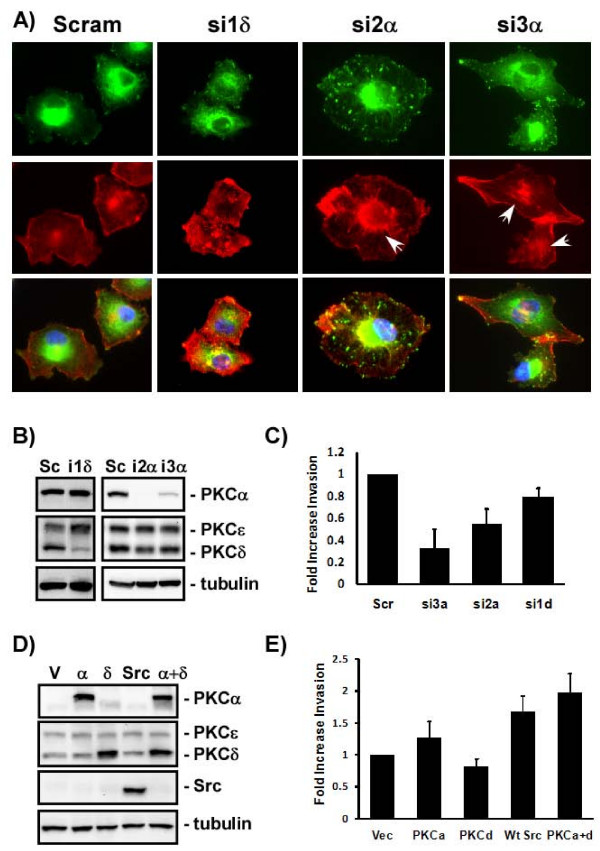
**Both PKCα and PKCδ are required for αvβ3-mediated invasion**. A) C8161.9 cells were transfected with scrambled (Scram), PKCδ siRNA (si1δ), or two PKCα siRNAs (si2α, si3α) and siRNA-transfected cells were plated on vitronectin for 2 hours and the extent of stress fiber and focal adhesion formation was monitored by staining with phalloidin (red) and immunostaining with anti-paxillin (green) respectively. Nuclei were counterstained with Hoescht (blue). White arrows indicate actin stress fibers around the perinuclear area in PKCα siRNA-treated cells. B) C8161.9 cells were transfected with scrambled (Sc), PKCδ (i1δ), si2α PKCα (i2α), or si3α PKCα (i3α) siRNAs. The levels of PKCα, PKCδ, and PKCε in siRNA transfected cells were measured by immunoblotting with isoform-specific PKC antibodies. Total levels of tubulin were also monitored (tubulin). C) C8161.9 cells were transfected with scrambled (Sc), PKCα siRNAs si3α (si3a), si2α (si2a), or PKCδ siRNA (si1d) and tested for their ability to invade VN-enriched Matrigel. Data represents at least 4 independent experiments. D) M14 cells were infected with viruses expressing nothing (V), PKCα (α), PKCδ (δ), Src, or PKCα and PKCδ (α + δ). Total levels of each protein were monitored by immunoblotting. E) Ability of the over expressing M14 cells to invade VN-enriched Matrigel was monitored. Data represents 3 independent experiments.

Inhibition of Src was sufficient to induce stress fibers and increase focal adhesion formation in C8161.9 cells adherent to VN (see Figure 3C). Inhibition of PKCα expression by siRNA increased focal adhesion formation, but only partially restored stress fibers – mostly around the nucleus (Figure 6A). Inhibition of PKCδ primarily increased stress fibers, with some increase in the size, but not number of focal adhesions. However, neither response was as strong as seen with inhibition of Src (see Figure 3C). Thus PKCα appears to more strongly influence focal adhesions, while PKCδ primarily targets stress fibers.

To delineate which PKC isoform is required for αvβ3-mediated melanoma invasion, C8161.9 cells were transfected with scrambled or PKC isoform-specific siRNAs to inhibit PKC expression (Figure 6B) and tested for their ability to invade VN-enriched Matrigel. Loss of PKCδ resulted in a slight (20%), but consistently reproducible decrease in invasion (Figure 6C). Loss of PKCα resulted in a 2-fold decrease in Matrigel invasion (Figure 6C).

Src, PKCα, or PKCδ was over expressed in M14 cells to determine if Src and PKC are sufficient to convert αvβ3-expressing M14 cells to an invasive phenotype (Figure 6D). Over expression of PKCα or PKCδ alone was not sufficient to induce M14 cell invasion of VN-enriched Matrigel (Figure 6E). However, over expression of Src alone or both PKCα and PKCδ increased M14 invasiveness 1.6- and 2-fold respectively.

## Discussion

Increased expression of αvβ3 integrin correlates with increased invasiveness and metastasis of melanoma *in vivo *[2]. Here we demonstrate that αvβ3 integrin expressed on the cell surface is not sufficient to generate invasive cells. Normal cultured primary melanocytes and one melanoma cell line both express αvβ3, yet they are not invasive. Efficient αvβ3-mediated invasion of the highly metastatic melanoma cell line C8161.9 requires at least two additional biochemical alterations; elevated Src activity and elevated expression of PKCα and PKCδ. In addition, over expression of Src or co-expression of PKCα and PKCδ is sufficient to convert the non-invasive αvβ3 integrin-expressing M14 cells to an invasive phenotype.

αvβ3 integrin binding to its vitronectin ligand is known to activate both PKC and Src [11,45]. So why would invasive melanoma cells require more Src and/or PKC activity if αvβ3 integrin is sufficient to activate these two pathways? It is possible that the sustained activity of PKC and/or Src signaling (and their inability to be turned off) is necessary to keep αvβ3 integrin signaling turned on long enough for effective invasion. The β3 integrin subunit is known to undergo a conformational change that enhances cell adhesion via mechanism termed 'inside-out' signaling and PKC has been shown to be important for αIIbβ3 activation in platelets [46]. An antibody that recognizes the activated form of β3 integrin has been described [47]. However, we failed to detect any difference in αvβ3 integrin activation state between C1861.9 or M14 cells using this antibody (data not shown). Thus increased signaling through PKC and Src does not increase invasiveness by altering inside-out signaling in these cells. Another possibility is that sustained signaling is required for enhanced secretion and activation of matrix metalloproteases (MMPs) required for movement through the ECM. Both Src and PKC are known to control MMP activity, and C8161.9 cells are known to have high levels of MMP activity [48], while M14 cells have low activity [49]. Conversely, elevated Src and PKC activity alone would not be sufficient for invasion, because the cells need the integrin to engage the substrate and localize proteases to facilitate movement.

At first glance it would appear that our findings contradict previous studies in which αvβ3 integrin expression in αvβ3 negative non-invasive melanomas was sufficient to generate invasive melanomas [5]. However, the status of PKC and Src signaling in those cells was not evaluated. Thus, it is possible that PKC or Src were already elevated or active in those cells and the addition of αvβ3 was all they needed. Another possibility is that because the αvβ3 reconstitution assays were carried out under serum and growth factor stimulatory conditions (which was absent from our in vitro assays), the presence of exogenous factors were sufficient to generate the PKC and/or Src signaling events needed to sustain αvβ3-mediated invasion.

Our studies have focused only on αvβ3 integrin and its ligand vitronectin. We can't rule out the possibility that other integrins binding other matrices might work just as effectively at promoting invasion in the context of elevated PKC and Src signaling. C8161.9 cells will not invade Matrigel (primarily a laminin-based substrate) that has not been treated with vitronectin. Thus laminin integrins are not sufficient for invasion even when PKC levels and Src activity are elevated. C8161.9 cells will invade Matrigel that has been treated with fibronectin (data not shown). We have not determined if this is solely due αvβ3 or whether another RGD integrin such as α5β1 is also involved. Others have reported that α5β1 integrin can regulate melanoma invasion on fibronectin [48]; however, the signaling pathways involved in α5β1-dependent invasion were not investigated. In the complex microenvironment found within a tumor-containing tissue, it is quite possible that several integrins may function together to facilitate invasion. We have delineated at least one possible mechanism by which αvβ3 integrin bound to its primary ligand vitronectin facilitates invasion.

In vivo studies demonstrating notable changes in Src expression levels or activity have rarely been reported in human melanoma samples. Nonetheless, increased Src kinase levels and activity in several cultured melanoma cell lines have been frequently reported and Src activity was shown to contribute to the formation of invasive posodomal structures that are prominently seen *in vivo *in melanoma samples [18]. That Src may contribute to αvβ3-mediated invasion and metastasis is not surprising given the known role that Src has been shown to play in many integrin-dependent events. Our ability to cause non-invasive melanoma cells to become invasive by Src over expression and to reduce invasion of highly invasive cells by inhibiting Src, was also observed in a study comparing the highly metastatic K1735M2 melanoma cells to the poorly metastatic K1735C23 [19]. In the K1735 cells invasion was linked to αvβ3 integrin expression and FAK activation, a known Src substrate. We have found that another effect of increased Src signaling in metastatic melanoma cells is to suppress RhoA activity, through Src-dependent phosphorylation and activation of one of Rho's negative regulators, p190RhoGAP.

Our results strongly support the previous finding that PKCα over expression is important for melanoma metastasis *in vivo *[21] and that loss of PKCδ suppresses melanoma metastatic potential [25]. In addition, elevated PKCα and PKCδ levels have been detected in metastatic melanoma cells taken from lymph nodes of patients [50,51]. Here we demonstrate that the high expression of PKCα, but not PKCδ, in C8161.9 human melanoma cells facilitates αvβ3-dependent invasion through regulation of Rac and activation of PAK and limiting focal adhesion formation. PKCδ, on the other hand, appears to limit stress fiber formation.

The link between integrins, PKC, and Rac activation is a new finding. However, a recent study demonstrated that syndecan 4-dependent cell migration was dependent on the ability of PKCα to activate Rac [52]. As in our system, this was unique to PKCα, in that loss of PKCδ or PKCε had no effect on Rac. The mechanism by PKCα regulates Rac activity has yet to be determined, but may involve regulation of Rac GEFs or GAPs. Two other Rac/PKC relationships have recently been reported. In colon cancer cells Rac signaling to PAK controls PKC/fascin interactions to regulate their migration, and spingosine1P signaling to PKCε subsequently activates a PLD2-PKCζ-Rac1 cascade to stimulate migration of endothelial cells [53,54]. Thus there may be several ways to interface PKC and Rac signaling to regulate motility in cells.

Even though PKCδ was not involved in regulating Rac/PAK signaling in our melanoma cells, it nonetheless contributes to efficient invasion. Loss of PKCδ only partially blocks invasion of C8161.9 cells, while over expression of both PKCα and PKCδ in non-invasive M14 cells was required to induce invasion. The differential ability of PKCδ and PKCα to regulate stress fibers versus focal adhesion formation respectively indicates that each PKC isoform contributes differently to actin remodeling, which is consistent with differential affects of each PKC isoform on Rac activation and the need for both isoforms to induce M14 cell invasion. Src has the ability to control both stress fibers and focal adhesions and is sufficient to induce invasion of M14 cells. The inability to completely restore stress fibers and focal adhesion upon inhibition of PKCs suggests Src is still able to exert some of its effects. Conversely, loss of Src alone is not sufficient to completely inhibit invasion of the C8161.9 cells, presumably because PKCs are still active. Together our data suggest some level of cooperation between PKCs and Src in optimally enhancing invasion by balancing the formation of adhesion structures and regulation of motility through differential regulation of Rac and RhoA.

Our studies have focused on the small GTPases RhoA and Rac, because of their known involvement in regulating the specific actin arrangements we observed in C8161.9 cells. Gene expression analyses of metastatic melanoma have consistently identified RhoC as a major component of metastatic melanoma *in vivo *[55,56]. However, in isolated cell lines grown in culture, RhoC expression is rarely detected. Thus the unique microenvironment generated *in vivo *appears to favor RhoC expression, which has been demonstrated to be required for metastasis *in vivo*. We did not detect RhoC expression in C8161.9 cells and RhoC was not detected in our Rhotekin pull-down assays (data not shown). One study demonstrated that over expression of RhoC in a radial growth phase melanoma cell line, WM35, promoted cell invasion and increased signaling through the PI-3K/Akt pathway, independent of signaling through the RhoA downstream effector ROCK [57]. Whether the effects of RhoC on *in vitro *invasiveness or *in vivo *metastasis are linked to αvβ3 remains to be determined.

## Conclusion

Elevated expression levels of PKCα and PKCδ, as well as constitutively high Src activity in highly metastatic melanoma cells are critical for optimal αvβ3-dependent invasion. Over expression of Src or co-expression of PKCα and PKCδ is sufficient to convert αvβ3-expressing non-invasive melanoma cells to highly invasive cells. Src is responsible for suppressing αvβ3-dependent activation of RhoA via stimulation of p190RhoGAP, while PKCα is required for Rac-dependent activation of PAK. PKCα predominantly represses focal adhesion formation, while PKCδ represses stress fibers. The net effect of the combined action of PKC and Src is to increase the levels of Rac activity relative to RhoA, which inhibits stress fiber formation and reduces focal adhesion and cell attachment favoring a more migratory phenotype required for αvβ3-dependent invasion.

## Abbreviations

PKC: protein kinase C; ECM: extracellular matrix; VN: vitronectin; siRNA: small interfering RNA; TPA: 12-O-tetradecanoylphorbol 13-acetate; GFP: green fluorescent protein; BSA: bovine serum albumin; PBS: phosphate buffered saline; IgG: immunoglobulin; GAP: GTPase activating protein; GEF: guanine nucleotide exchange factor; PI-3K: phosphatidylinositol 3'-kinase.

## Competing interests

The authors declare that they have no competing interests.

## Authors' contributions

AP conceived of the study, participated in the design, carried out a significant portion of the experiments, and read and approved the final manuscript. VS participated in the design, carried out half the experiments, addressed the reviewers concerns, and helped draft the manuscript. EF was instrumental in making the connection between Src and Rho signaling. HB made the initial discovery of the connection between PC and Rac signaling. CM designed and coordinated the study, interpreted the data, and drafted the majority of the manuscript and incorporated the required revisions.
